# When in Rome, Do Like the Romans: Certifying Stroke Centers With the Rod of Aesculapius or the Medical Caduceus of Hermes?

**DOI:** 10.1161/JAHA.113.000120

**Published:** 2013-04-24

**Authors:** Lee H. Schwamm

**Affiliations:** 1 Department of Neurology, Massachusetts General Hospital, Boston, MA (L.H.S.)

**Keywords:** Editorials, acute stroke, cerebrovascular disease/stroke, emergency medical services, health policy and outcomes research, thrombolysis

## Introduction

One of the first hospitals in the Western world was the *Aesculapium* which was built in Rome along the Tiber Island in 293 bc and included a long‐term recovery center. When the emperor Claudius granted freedom to slaves who had been there for years, it was probably aimed more at disposition of the patients and recovery of the beds they occupied than emancipation.^1^ The symbols which reflect the historical tradition of the profession of medicine, and in turn identify for the public those places in which the art of medicine and the principles of Hippocrates are practiced, provide a compelling and relevant perspective.

Perhaps the oldest and best known such symbol is the Rod of Aesculapius. It was carried by Aesculapius, the son of Apollo and the Greco‐Roman god of medicine. When snakes were said to have licked clean the ears of the young Aesculapius, they taught him secret knowledge that led to him becoming a great healer. He carried a simple rod hewn of wood around which coiled a single serpent, and this rod became a prime symbol of the healing arts (Figure – Panel A). Asclepius died at Zeus' hand when he angered Hades by accepting gold in payment for resurrecting the dead.^2^ While the Rod of Aesculapius remains the predominant symbol of medicine around much of the world, starting in the 1900s in the US a different serpent‐themed symbol became equally popular. A double serpent‐entwined staff with surmounting wings is often designated as the “medical caduceus” and is derived from the double serpent‐entwined staff of Hermes, the Greco‐Roman god of commerce (Figure – Panel B). This modern caduceus became a popular medical symbol after its adoption by the US Army Medical Corps at the turn of the 20th century.^3^ A 1990 survey of US organizations found that professional associations were more likely to use the Rod of Aesculapius (62%). Hospitals (37%), though, were a notable exception, while commercial organizations were more likely to use the caduceus (76%).^4^ This tension between the art of healing and the commercial realities within modern medicine, so simply illustrated in these 2 conflicting symbols of medical care, provides a lens for examining the proper place and method of certification of stroke centers within an altruistic stroke system of care.

**Figure 1.**
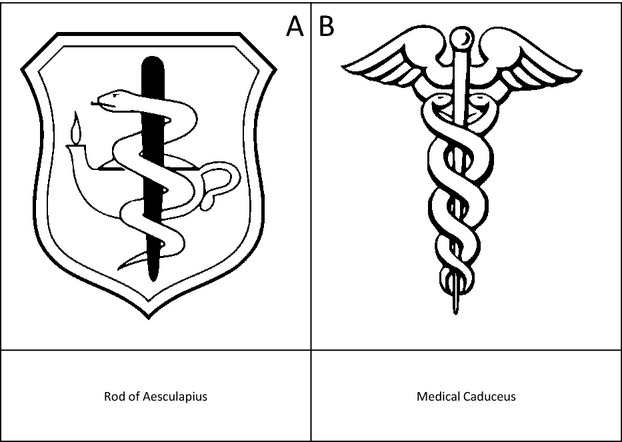
Graphic depictions of the medical symbols of the single serpent‐entwined wooden Rod of Aesculapius in this image superimposed on a lantern (A, on left), and the double serpent‐entwined staff with surmounting wings of the Medical Caduceus (B, on right).

In their article in this issue of the *JAHA*, Mullen et al^5^ report on an analysis of the use of intravenous thrombolysis and its association with Joint Commission (JC) primary stroke center (PSC) certification, which is potentially an important proxy for superior care delivered at PSC. They used the Nationwide Inpatient Sample to estimate the effect of a hospital's JC PSC status on the odds of its receiving intravenous tissue type plasminogen activator (IV tPA) in a population of over 300 000 ischemic stroke patients admitted directly to US hospitals in ≈25 states from 2004 to 2009. Over that period, they found that tPA administration increased from 1.4% to 3.3% at non‐PSCs and from 6.0% to 7.7% at PSCs. While being treated at a PSC increased the odds of receiving IV tPA (OR 1.87, 95% CI 1.62 to 2.17) and the association of certification and tPA utilization was significant in all years, the effect size was strongest in 2004 (OR=2.95) and weakest in 2009 (OR=1.68). This finding is important, and demonstrates that rates of tPA use are increasing, and that centers that are PSC‐certified are making a major contribution to the use of IV tPA. However, they also found that the proportion of patients treated with tPA at non‐PSCs more than doubled over the study period, while the proportion treated at PSCs remained stable. This raises the important question as to whether or not it is the PSC certification or other factors that are contributing to the difference. The authors speculate that hospitals without certification may have been preparing for future certification by participating in quality improvement initiatives such as Get with the Guidelines (GWTG), or by utilizing telestroke programs, both of which may lead to increased tPA utilization regardless of PSC status. All of these reasons are likely in play a role, and they raise the question of how many patients were already being treated at hospitals that would one day become 1 of the 1000 PSCs currently certified by the JC in the United States. Did the rates of tPA‐use continue to increase at these hospitals after they became PSCs compared to before certification? This seems unlikely given the data provided, but the NIS data are limited in helping to answer these important questions. As hospitals seek certification in the new JC comprehensive stroke center (CSC) program, the need for evidence will only grow.^6–7^

In 1996, despite the availability of an emergency medical services (EMS) system, modern ambulances, widespread access to CT imaging, and approval of tPA, the reality on the ground was that hospitals were ill‐equipped to provide this highly efficacious therapy. Experiences in the pivotal trials^8^ and acute myocardial infarction identified best practices, and in 1999 the American Heart Association/American Stroke Association (AHA/ASA) launched “Operation Stroke” which helped 1500 hospitals build infrastructure designed to meet JC certification requirements based on the Brain Attack Coalition's (BAC) recommendations for formation of PSCs.^9–10^ The Institute of Medicine^11–12^ released several influential reports on fragmentation in health care systems directly relevant to stroke care, and in 2005 the AHA responded with recommendations for the implementation of a model of stroke systems of care to encourage the coordinated delivery of care.^13^ A 2008 AHA survey (from Ranous J, AHA, unpublished data, personal communication, 2012) found that 66% of hospitals surveyed did not have stroke protocols, 82% did not have rapid identification for acute stroke patients, and many acute care hospitals lacked the necessary staff and equipment to provide optimal, safe, and effective emergency care for these patients and patients were waiting an average of 3 to 6 hours to seek treatment. EMS providers were not consistently trained to recognize stroke and less than 3% of patients were being treated with tPA.

At the center of the 2005 AHA recommendations^13^ for stroke systems of care is a set of principles governing regionalized systems of care. First and foremost, “a stroke system should ensure *effective interaction and collaboration* among the agencies, services, and people involved in providing prevention and the timely identification, transport, treatment, and rehabilitation of individual stroke patients in a locality or region. Second, a stroke system should promote the use of an *organized, standardized approach* in each facility and component of the system. Third, a stroke system should identify *performance measures* (both process and outcomes measures) and include a mechanism for *evaluating effectiveness* through which the entire system and its individual components continue to evolve and improve.”

A fundamental concept in this model is that measurement of performance, rather than just the presence of prescribed infrastructure or good intentions, should be the yardstick by which stroke centers and systems are evaluated. A consensus set of PSC stroke measures have been in use since 2005 (Table).^14^ It also articulated that care for acute stroke patients should be regionalized and that patients should be “transported to the nearest primary stroke center or hospital with an equivalent designation, given the available acute therapeutic interventions.” This must be tempered by the realities of primary stroke center geospatial distribution and transport times. This is because in the United States, these PSCs are not distributed according to need or population density, but are voluntary designations for which all US hospitals may compete. For many hospitals, the motivation to seek PSC designation is a blend of altruistic, financial, and strategic goals and this is not well aligned with the most efficient use of healthcare dollars to provide safe and equitable care to a population. In London, recent sweeping changes to the organization of hyperacute stroke care occurred with the designation of hyperacute stroke units by the public health authorities based on population data rather than prior experience or hospital interest, with these centers being strategically placed across the city of London.^15^ This strategy has produced a substantial increase in the use of thrombolysis in the city of London, but did so at the cost of substantial upheaval within the previous organically grown system of care.

**Table 1.** Comparison of Stroke Performance Measures by Stroke Subtype in the National Stroke Quality Programs of the CDC, GWTG, JC, and the NQF

Performance Measures	CDC	GWTG	JC	NQF
DVT prophylaxis	AIS, ICH/SAH	AIS, ICH/SAH, TIA*	AIS, ICH/SAH	AIS, ICH/SAH
Discharged on antithrombotic therapy	AIS, TIA	AIS, TIA*	AIS	AIS
Discharge on anticoagulation for patients with atrial fibrillation	AIS, TIA	AIS, TIA*	AIS	AIS
Thrombolytic therapy administered	AIS	AIS*	AIS	AIS
Antithrombotic therapy by the end of hospital day 2	AIS, TIA	AIS, TIA*	AIS	AIS
Discharged on cholesterol reducing medication	AIS, TIA	AIS, TIA*	AIS	AIS
Dysphagia screening	AIS, ICH/SAH	AIS, ICH/SAH	AIS, ICH/SAH	—
Stroke education	AIS, ICH/SAH, TIA	AIS, ICH/SAH, TIA*	AIS, ICH/SAH	AIS, ICH/SAH
Smoking cessation	AIS, ICH/SAH, TIA	AIS, ICH/SAH, TIA*	AIS, ICH/SAH	—
Assessed for rehabilitation	AIS, ICH/SAH	AIS, ICH/SAH*	AIS, ICH/SAH	AIS, ICH/SAH

CDC indicates Centers for Disease Control; GWTG, Get With the Guidelines‐Stroke; JC, Joint Commission; NQF, National Quality Forum; DVT, deep vein thrombosis; AIS, acute ischemic stroke; ICH, intracerebral hemorrhage; SAH, subarachnoid hemorrhage; TIA, transient ischemic attack.

* The 7 GWTG achievement measures used for hospital recognition programs.

Adapted from Reeves et al.^14^

Transparency for the public and providers is critical to ensure safe, equitable care and the public trust. Hospital or corporate affiliations, as well as local and state boundaries, should not interfere with the safe and efficient care and transport of stroke patients. A stroke system should determine the acute stroke treatment capabilities and limitations of all hospitals and make these available to primary care providers, EMS, and the public. Hospitals that are identified as “acute stroke capable,” should be monitored to ensure that they possess the appropriate resources in accordance with national recommendations and local or national certifying bodies. Hospital certification, designation, or licensure may be accomplished through a variety of organizations (eg, nonprofit companies, state health agencies, professional societies, or JC) based on these national recommendations. But having the resources alone is not sufficient. Certification should also include monitoring of the quality of care actually delivered. While the current JC program for certification of PSC does an excellent job of assessing the resources, protocols, and infrastructure of candidate hospitals, the program could go much further. To support an accountable system of stroke care, it should require hospitals to achieve a prespecified level of achievement in order to remain recognized.

The AHA/ASA's GWTG‐Stroke is a quality measurement and improvement program developed to increase the quality of stroke care through data collection, patient‐specific guideline recommendations, real‐time data validation, and tracking of adherence to the guidelines individually and against national benchmarks. Hospitals using GWTG‐Stroke that achieve adherence to 7 evidence‐based practices in greater than 85% of all eligible cases are recognized with awards. Since 2003, GWTG‐Stroke has been implemented in more than 2000 US hospitals and collected ≈2.5 million patient records. In contrast to the PSC program which is administered by the JC in partnership with AHA, the GWTG‐Stroke program does not incorporate on‐site inspections or rigorous work process and infrastructure standards. Ultimately, what is in the best interest of patients would be a hybrid of the 2 programs, whereby hospitals are recognized for participation but only receive true certification and designation if they achieve objective performance benchmarks. This is particularly important in regions where EMS agencies preferentially route acute stroke patients to certified PSCs. Since many hospitals with limited stroke resources are required to have formal transfer agreements with nearby PSC or CSC facilities, it is imperative that providers and the public believe that PSCs and CSCs deliver care that is measurably superior to that provided in noncertified community or teaching hospitals.

Hard evidence for superior health outcomes due to care at PSCs is emerging but limited. Reports from hospitals in Europe have shown that the presence of stroke units, a hallmark of the PSC guidelines, is associated with better outcomes.^16–17^ These data are frequently cited as evidence that US PSCs improve care. However, much of the benefit in stroke unit care may be attributable to the implementation of aggressive early rehabilitation and mobilization that is the hallmark of these European units and is lacking in most US PSCs where the length of stay for many patients is often less than 5 days. A study to evaluate the effectiveness of PSCs compared unadjusted and risk‐adjusted 30‐day mortality and readmission rates of elderly patients with ischemic stroke treated at hospitals that would become PSC‐certified within the first few years of the program with those treated at hospitals that did not subsequently become certified within the same period.^18^ The results revealed that JC PSC‐certified hospitals had better outcomes than noncertified hospitals even before the program began. A recent Finnish study found admission to hospitals that met criteria for PSC or CSC certification was associated with lower 1‐year case fatality and reduced institutional care compared with general hospitals.

What do we know about the performance of hospitals certified by other organizations? DNV Healthcare is part of Det Norske Veritas, and since 2008 has been licensed by the Centers for Medicare & Medicaid Services (CMS) to accredit acute care and critical access hospitals, and also provides PSC and CSC certification. No data are currently published on the performance of DNV accredited sites. Many states offer alternative PSC or equivalent designations for hospitals based on self‐designation or a program administered by the State Departments of Public Health (DPH). A New York DPH analysis reported better outcomes in state‐designated PSCs that met all Brain Attack Coalition (BAC) PSC requirements.^19^ Admission to designated centers was associated with modestly greater use of thrombolytic therapy and lower all‐cause mortality at 1‐, 7‐, 30‐day and 1‐year follow‐up. By contrast, the Primary Stroke Service (PSS) designation was implemented by the Massachusetts DPH as a data‐driven alternative to PSC designation. It is based on the BAC criteria focused on tPA delivery, and required evidence of acute stroke teams, 24‐hour rapid brain imaging and interpretation, neurosurgery coverage within <2 hours of request (including by transfer), mandatory stroke registry data collection, performance review, community education, and an on‐site verification by DPH personnel (data form available at http://www.mass.gov/eohhs/docs/dph/quality/healthcare/stroke-data-form.pdf). By December 2005, 92% of Massachusetts hospitals achieved PSS designation, and only one of these pursued JC certification.^20^ Prospectively acquired data were analyzed on consecutive acute stroke patients arriving ≤3 hours after stroke onset at all 69 PSS sites between 2004 and 2008, many of whom also participated in GWTG‐Stroke. IV tPA was given to 22.1% of patients arriving ≤2 hours and increased steadily from 2005 to 2008 in these patients (18.4%, versus 25.5%; *P*<0.001) and in all ischemic stroke discharges (6.7% versus 10.4%, *P*=0.0001).^21^ This increase among hospitals that were not certified PSCs compares favorably to the rates reported by Mullen et al. Patients who arrived at MA GWTG‐participating hospitals were more likely to receive IV tPA after versus before that hospital achieved award recognition for its performance on all 7 indicators (28.1% versus 22.3%, *P*<0.001), and tPA was more likely to be given at award hospitals (32% versus 20%, *P*<0.001). When award status was added to the multivariable regression model, it was independently associated with IV tPA use with the highest odds ratio (adjusted OR, 1.4; 95% CI, 1.1 to 1.7).

What seems clear is that the advent of stroke center certification has been driving hospitals and public health authorities to increasingly view stroke from a broader perspective, and the rapid adoption of certification has undoubtedly raised the bar on stroke care in the United States. With the announcement of CSC certification, a new phase of certification is beginning. Whether or not “Stroke Center Certification 2.0” will lead to better care and more effective regionalization versus silos of care and polarization, is uncertain. The data reviewed to date provide compelling evidence that PSC certification alone, or quality improvement programs alone, without the critical accompanying aspect of measurement and accountability, may be insufficient to drive improvements in health outcomes for stroke patients. In an era of increasing pressure on healthcare expenditures and unprecedented mergers of hospitals and providers, we should seek to set aside the medical caduceus in favor of the Rod of Aesculapius, and design stroke systems of care that will adhere to the principles laid down in 2005. Namely, these systems should strive to provide access to high quality acute stroke care to all citizens regardless of their geographic location, emphasize public–private partnerships and mandatory reporting of objective measurable performance criteria, and reduce the risks inherent in the multiple transitions of care experienced by stroke patients. If we are so fortunate, perhaps the serpent of Aesculapius will lick clean our ears so that we can be taught the secret knowledge needed to design the next iteration of stroke care that will radically improve the health outcomes of our patients.
